# The Prevalence of Stunting and Associated Factors among Children Under Five years of age in Southern Ethiopia: Community Based Cross-Sectional Study

**DOI:** 10.5334/aogh.3432

**Published:** 2021-11-17

**Authors:** Asres Mengesha, Samrawit Hailu, Mahlet Birhane, Moges Mareg Belay

**Affiliations:** 1 Department of Reproductive health, School of public health, College of Medicine and Health Sciences, Dilla University, Dilla, Ethiopia; 2 Department of Public health, School of public health, College of Medicine and Health Sciences, Dilla University, Dilla, Ethiopia; 3 Department of Human Nutrition, School of public health, College of Medicine and health Sciences, Dilla University, Dilla, Ethiopia

## Abstract

**Background:**

According to the Ethiopian Mini Demographic Health survey (EMDHS) of 2019, about 37% of children under five years of age are stunted. Data are scarce on stunting in the study area.

**Objective:**

This study was aimed to assess the prevalence and factors associated with stunting in among children under five years of age in southern Ethiopia.

**Method:**

A community-based cross-sectional study was conducted among 660 randomly selected under five child-mother pairs. The study was conducted from December 1 to 30, 2018 using a structured pretested questionnaire and anthropometric measurement tools. A simple random sampling technique was used to select study participants. Data were entered into EpiData version 3.1 and analyzed by Statistical Package for the Social Sciences (SPSS) version 20 and Emergency Nutrition Assessment (ENA) for Standardizing Monitoring and Assessment of Relief and Transition (SMART) 2011 software. Variables with P-value < 0.25 during the bivariate were entered into multivariable logistic regression analysis and significant association with stunting was declared at P-value < 0.05 with 95% CI.

**Result:**

Prevalence of stunting among children under five years of age was 37.7%. Factors: family size less than five [AOR = 0.59; 95% CI (0.37, 0.97)], age less than 11 months [AOR = 0.17; 95% CI (0.08, 0.4)] and rich wealth status [AOR = 0.46; 95% CI (0.27, 0.79)] had a protective effect, while source of drinking water like river water [AOR = 5.11; 95% CI (1.6, 16.4)], presence of two or more under five children in the household [AOR = 1.72; 95% CI (1.07, 2.77)], undiversified diet [AOR = 1.82; (1.17, 2.83)] and household food insecurity [AOR = 1.83; 95% CI (1.13, 2.96)] increased the risk of stunting.

**Conclusion and recommendation:**

The prevalence of stunting was high. Child age, family size, number of children under five years of age in the household, wealth status, source of drinking water, undiversified diet, and household food insecurity were associated with stunting. Thus, efforts should be made to improve nutritional status through strengthening of nutrition education, promotion of different family planning methods to limit the family size, involvment in different income generating activities to improve wealth status, securing of household food, use of improved sources of water, and nutrition education to diversify child diet. Further longitudinal study is recommended for researchers.

## Introduction

Stunting shows a failure to achieve one’s genetic potential for height and is caused by inadequate nutrition during infancy [1,2]. Because this developmental phase does not reoccur in later life, reversing or treating this condition later in childhood is almost impossible even within an improved environment [3]. The long-term consequences of stunting include reduced school performance, low work productivity, and adverse pregnancy outcomes. Worldwide, stunting affects nearly one-third of children under five years of age, with a higher prevalence reported in low-resource countries of sub-Saharan Africa and South Asia [4,5]. Stunting in young children is the result of various factors, comprising pre-pregnancy, intrauterine, and postnatal malnutrition. More commonly, stunting can be caused by insufficient or inappropriate nutrition and the impact of infectious disease [6,7].

Globally, it is estimated that undernutrition is responsible, directly or indirectly, for at least 35% of deaths in children under five years of age. It affects nearly 195 million children under five years of age in developing nations [8]. Worldwide, one out of four children, one-third of children under five years of age in low and middle-income countries—and in Ethiopia, more than 2 out of every 5 children—are estimated to be stunted [9,10,11]. Ethiopia is the second-most populous country in Africa, with 15.4% of children under five years of age [12]. Malnutrition is the underlying cause of 57% of child deaths in Ethiopia [13], with some of the highest rate of stunting and appearance of underweight children in the world [14].

Sixty seven (67%) of the working-age population in Ethiopia is currently stunted with, on average, lower school levels than those who did not experience growth retardation by 1.1 years of lower schooling [11].

According to the latest EMDHS of 2019, in Ethiopia, 37% of children under five years of age were stunted, and 12% were severely stunted. The survey also revealed that in the Southern Nations Nationalities and People Region (SNNPR) of Ethiopia the percentage of children under five years of age who were stunted or severely stunted was 36.3% and 12.4%, respectively [15].

To achieve the goals of accelerated stunting reduction, identifying the potential determinants of chronic undernutrition is a vital step. Despite many studies conducted at national and regional levels, the prevalence and risk factors at sub-regional or community levels have been insufficiently emphasized, particularly at the Gedeo zone, which makes interventions difficult in such circumstances. Thus, it is important to have detailed and concrete data that can fill these gaps and add value that directs policymakers to draw appropriate intervention measures to improve and flourish the health of a future generation. Therefore, this study investigated the prevalence and factors contributing to stunting among children under five years of age in the Wonago district, Gedeo zone, southern Ethiopia.

## Materials and methods

### Study setting and period

A community-based cross-sectional study was conducted in Wonago district, Gedeo zone, Southern Ethiopia from December 1 to December 30, 2018. The district is located 100 kilometers South of Hawassa, capital of Southern Nations, Nationalities, and Peoples’ Regional State, and 14 kilometers South of Dilla, which is the capital of the Gedeo zone. In 2018, the district had 31,893 households with a total population of 156,274. The total number of children under five years of age in the woreda is 24,394 [16]. According to the Wonago district health office report of 2018, Wonago district has 21 kebeles (smallest administrative units in Ethiopia’s government adminstration), with 6 health centers, 20 health posts, and 2 private clinics. The source population was all children under five years of age with their caretakers in Wonago district. Children under five years of age with their caretakers in selected kebeles of Wonago district were study participants. Children under five years of age from families who have lived at least six months in the Wonago district were included in this study.

### Sample size determination and sampling procedures

The sample size was calculated by using single a population proportion formula by considering the following assumptions, where n = required sample size, Z = critical value for normal distribution at 95% confidence level (1.96), P = 26.6% of children under the age of five years are stunted [17] and d = 0.05 (5% proportion of tolerable sampling error between the sample and the population).

n = \frac{{{Z^2} * p * \left({1 - p} \right)}}{{{d^2}}} = \frac{{{{\left({1.96} \right)}^2} * 0.266 * \left({1 - 0.266} \right)}}{{{{\left({0.05} \right)}^2}}} \approx 300

As a multistage sampling technique was employed to identify study subjects, a design effect of 2 was used. Adding 10% of the total sample size to compensate for the non-response rate, the final sample size was 660.

Multi-stage sampling was employed to include study participants. At the first stage of sampling, from a total of twenty-one kebeles that are found in the district, seven kebeles were selected by using a simple random sampling technique considering one-third of the total kebeles. In the second stage, a total of 5,221 households that have at least one child below five years of age paired with their caretakers was obtained from the health post family folder of the selected kebeles with the help of health extension workers. The number of eligible households was allocated by using population proportional allocation. When two or more eligible children were found in the same household, the youngest one was selected to minimize recall bias. When the eligible households were closed during data collection, they were revisited, and if they were found still closed at the second visit, they were considered as non-respondents.

### Data collection tools and techniques

The study used a close ended questionnaire, which was adapted from EDHS 2011 and other literature. It was pre-tested and administered to mothers/caregivers by interviewers during data collection. The questionnaire was first contextualized and developed in English and then translated into the local language (Gede’uffa). Then it was translated back to English to assure its consistency. Data were collected by individuals who had completed their secondary education and have trained on different data collection techniques by NGOs. Two health officers were recruited for supervision.

Anthropometric data were collected using the standard procedure determined by the World Health Organization (WHO) [18] using instruments such as a wooden length board, a vertical wooden height board with a detachable sliding headpiece (designed by the United Nations Children’s Emergency Fund [UNICEF]), and a Mid Upper Arm Circumference of the Child (MUAC) measuring tape. The body length of under-two children was measured with bare feet by using a horizontal wooden length board with the infant in a recumbent position. However, the height of children 24 months and above was measured using a vertical wooden height board by placing the child on the measuring board and letting the child stand upright in the middle of the board. Length/height was taken to the nearest 1 cm. Children were defined as stunted if their height-for-age is more than two standard deviations below the WHO Child Growth Standards median.

### Data quality control

Training was given to data collectors and supervisors before data collection. Anthropometric measurements were also demonstrated. The pretest was done on 5% of the sample at another kebele of Wonago district—one not selected for data collection—and some modification was made. The weight scale was calibrated to zero reading before and after weighing every child. To determine the accurate age of the child, every effort has been made by observing birth certificates, immunization cards, and asking the memory of special events in the mother’s or caretakers’ life. Maternal factors were assessed by interviewing only the biological mothers of the child, which could minimize potential recall biases. Double data entry and validation were carried out.

### Data processing and analysis

Data were coded and entered into EpiData version 3.1, then exported to SPSS version 20 software for cleaning and analysis. Anthropometric data were exported to Emergency Nutrition Assessment (ENA) 2011 software for analysis and again exported to Microsoft Excel to have meaningful consistency. Then analyzed data of anthropometry was exported back to SPSS data.

Principal component analysis (PCA) has been carried out by the reduction of variables involved in the development of wealth, access to diversified diet, and household food security statuses. Principal component analysis (PCA) was carried out using 15 variables. Twelve of the variables were binary, while three variables were recoded to a meaningful variable. In this study it was measured by giving a score of “1” for possessing each of 12 items in the list, and those two variables having a different scoring system were used according to their value. The summed items were then classified into tertials (poor, medium, and rich).

A total of 12 food group items assessing household food diversity level; and a total of 9 frequency of occurrence questions among 18 household food insecurity access scale generic questions that appear to distinguish the food secure from unsecured households were dimensionally reduced to single access to diversified diet variable and household food security variable respectively.

Among the variables that had been entered into the pool of PCA, components whose Eigenvalue loads were greater than one were extracted. Factor one has been considered to address involved components. Since there are no established cut-off points in terms of food groups to indicate adequate or inadequate dietary diversity for the household and given that this can be analyzed in several ways [19], household access to diversified diet would be measured based on factor analysis, and the rank would be assigned into two categories from lowest to highest values; 1 was given for poor access and 2 for good access.

Similarly, the household food security status would be analyzed by factor analysis, and the rank would be assigned into two categories from highest to lowest values; so that 1 was given for food unsecured households and 2 for food secured households.

Descriptive statistics were displayed by using frequency, percentage, mean, and standard deviation. Binary logistic regression analysis was done to show the association between dependent and independent predictors. Variables with *P*-value < 0.25 in the bivariable binary logistic regression analysis were moved to multivariable logistic regression analysis. *P*-value < 0.05 was considered statistically significant in multivariable logistic regression. Finally, we estimated a measure of association by computing multivariable logistic regression models and reporting both adjusted odds ratios (AOR) and 95% confidence intervals (CI).

### The ethical standard of disclosure

This study was conducted per the Declaration of Helsinki. The study was approved by the Ethical Review Board (IRB) of Dilla University, College of Health Science and Medicine. The participants were informed about the purpose of the study and, to maintain confidentiality, the names of the respondents were not recorded. The right is also given to the study participants to discontinue the participation at any time during the interview. Finally, data were collected after obtaining written informed consent from the study participants.

## Results

### Socio-demographic characteristics

A total of 615 children paired with 539 (87.6%) caretakers from rural and 76 (12.4%) from urban settings participated in the study, yielding a response rate of 93.2%. The majority of the respondents 487 (79.2%) were female, out of which 412 (67%) were biological mothers of the child. A greater proportion of the respondents—434 (70.6%)—were Gedeo in ethnicity, 512 (83.3%) of the respondents were protestant in religion, 336 (54.6%) were housewives in occupation, and 245 (39.8%) attended primary education level. The highest proportions of the households (570 [92.7%]) were headed by fathers. More than half (382 [62.1%]) of the households had a family size of five or more, and 346 (56.3%) had only one child under the age five years. Analysis of the wealth index of the respondents showed that the number of poor and those in a medium level of economic status were almost proportional: 222 (36.1%) and 227 (36.9%) respectively (***Table 1***).

**Table 1:** Socio-demographic and economic characteristics of the respondents in Wonago district, Gedeo Zone, southern Ethiopia, 2018 (n = 615).


VARIABLES	FREQUENCY	PERCENT (%)

**Caretaker’s relation with the child**		

Mother	412	67

Father	119	19.3

Other relatives	84	13.7

**Ethnicity of caretaker**		

Gedeo	434	70.6

Oromo	87	14.1

Amhara	55	8.9

Others	39	6.3

**The religion of the caretaker**		

Protestant	512	83.3

Orthodox	83	13.5

Muslim	16	2.6

Others*	4	0.7

**Head of the household**		

Father	570	92.7

Mother	35	5.7

Others£	10	1.6

**Educational status of the caretaker**		

No formal education	161	26.2

Primary education	245	39.8

Secondary	117	19

Higher-level	92	15

**Occupation of the caretaker**		

Housewives	336	54.6

Government/private		

employee	32	5.2

Merchant	146	23.7

Daily laborer	44	7.2

Farmer	30	4.9

Others^¥^	27	4.4

**Age of the caretaker**		

<20	3	0.5

20–34	421	68.5

35–49	172	28

>49	19	3.1

Marital status of the caretaker		

Single	13	2.1

Married	565	91.9

Divorced	8	1.3

Widowed	29	4.7

**Family size**		

< 5	233	37.9

≥ 5	382	62.1

**Number of under-five children in the household**		

One	346	56.3

≥2	269	43.7

**Wealth status of the family**		

Poor	222	36.1

Medium	227	36.9

Rich	166	27


* Catholic, traditional followers… ^£^ *Grandparents, other relatives* ^¥^ *Barbers, carpenter*.

### Child-related factors

The mean (±SD) age of children was 25.6 ±14.3 months and 327 (53.2%) of them were female. Less than half (43.7%) of children under five years of age had first birth order. The birth interval was assessed only for children beyond first birth order (346), so that there is a one year birth interval between 113 (32.6%) of the children under five years of age and their preceding children under five years of age. Similarly, it was 3 years among 113 (32.6%) of children under five years of age. Out of the 162 children below or 12 months of age, 69 (42.6%) of them had received vaccination in line with their age. Among 525 children above or 9 months age, 219 (41.7%) were fully vaccinated. One hundred fourteen children under five years of age (18.5%) had a history of comorbidities (***Table 2***).

**Table 2:** Child factors among children under five years of are in Wonago district, Gedeo Zone, southern Ethiopia, 2018 (n = 615).


VARIABLES	FREQUENCY	PERCENT (%)

**Age of the child**		

<6 months	72	11.7

6–11 months	74	12

12–24 months	151	24.6

>24 months	318	51.7

**Sex of the child**		

Male	288	46.8

Female	327	53.2

**Birth order of the child**		

First	269	43.7

Second	135	22

Third	109	17.7

Fourth or more	102	16.6

**The birth interval between the index and preceding child (n = 346)**		

1 year	113	32.6

2 years	67	19.4

≥3 years	113	32.6

I don’t know	53	15.4

**Receive vaccination in line with his/her age (n = 162)**		

No	55	33.9

Yes	69	42.6

I don’t know	38	23.5

**Fully vaccinated (n = 525)**		

No	134	25.5

Yes	219	41.7

I don’t know	172	32.8

**Deworming (n = 318)**		

No	139	43.7

Yes	122	38.4

I don’t know	57	17.9

**Child experience comorbidities**		

No	501	81.5

Yes	114	18.5


### Maternal factors

Out of the total 412 biological mothers, 117 (28.4%) of them did not have antenatal care (ANC) visits, 147 (35.7%) of the mothers followed ANC visits 1 to 3 times, 148 (35.9%) visited ANC more than 4 times. Regarding postnatal care (PNC), 133 (32.3%) of them attended PNC, and 123 (29.9%) of the respondents utilized family planning before the gestation of the index child.

### Feeding characteristics of children

Out of 412 children under five years of age from biologic mothers who were assessed for feeding status, 316 (76.7%) had feed colostrum. Out of 346 children under five years of age assessed for breastfeeding duration (≥24 months children), 177 (51.2%) fed on breast milk for less than two years and 169 (48.8%) fed for greater than or equal to two years. Concerning initiation of supplementary food, 217 (35.3%) of children under five years of age got supplementary feeding at six months, while 127 (20.7%) of them began before six months, and 69 (11.2%) of them began later than six months of life. Access to diversified diet and food security status of beyond half of the households in this study area were found to be good 342 (55.6%) and secured 370 (60.2%) respectively (***Table 3***).

**Table 3:** Feeding and dietary characteristics of children under five years of age in Wonago district, Gedeo Zone, southern Ethiopia, 2018 (n = 615).


VARIABLES	FREQUENCY	PERCENT (%)

**What was done to the colostrum (n = 412)**		

Given to the child	316	76.7

Withdrawn	96	23.3

**Duration of breast feeding (n = 346)**		

< 24 months	177	51.2

≥ 24 months	169	48.8

**When the complementary feeding was initiated?**		

Not given b/se child is <6 months age	46	7.5

Right at 6 months	217	35.3

After 6 months	69	11.2

I don’t know	130	21.1

**Frequency of child feeding per day (n = 543)**		

≤3 times	129	23.8

>3 times	304	56

I don’t know	110	20.2

**Access to a diversified diet**		

Poor	273	44.4

Good	342	55.6

**Household food security status**		

Food insecure	245	39.8

Food secured	370	60.2


### Personal hygiene and environmental sanitation characteristics

The main source of drinking water for the 277 (45%) of the respondents was public tap. The majority of the respondents 479 (77.9%) did not treat water, and 461 (75%) washed their hands before feeding their child. Of the total, 580 (94.3%) of the respondents had a latrine, out of which 544 (88.5%) were pit latrines. Regarding the disposal mechanism of domestic wastes, 279 (45.4%) of the respondents disposed domestic waste at an open field (***Table 4***).

**Table 4:** Personal hygiene and environmental sanitation characteristics among children under five years of age in Wonago district, Gedeo Zone, southern Ethiopia, 2018 (n = 615).


VARIABLES	FREQUENCY	PERCENT (%)

**The main source of drinking water**		

Public tap	277	45

Spring	222	36.1

Pond	51	8.3

Well	24	3.9

Private pipe	22	3.6

River	19	3.1

**Distance to drinking water**		

Less than 30 minutes	356	57.9

Greater or equal to 30 minutes	186	30.2

Water on-premise	73	11.9

**Treat water before drinking**		

No	479	77.9

Yes	136	22.1

**What type of equipment do you use to store water?**		

Jerrycan	366	59.5

Bucket	163	26.5

Pot	64	10.4

Others^@^	22	3.6

**Wash hand before feeding child**		

Yes	461	75

No	154	25

**Have latrine**		

Yes	580	94.3

No	35	5.4

**Type of latrine (n = 580)**		

Pit latrine	544	93.8

Ventilated improved latrine	28	4.8

Flush toilet	8	1.4

**Wash hand after latrine**		

No	564	91.7

Yes	51	8.3

**Share latrine with another household**		

No	580	94.3

Yes	35	5.7

**Disposal mechanism of domestic wastes**		

At open field	279	45.4

At pit	117	19

By municipality	73	11.9

By composting	69	11.2

By burning	77	12.5


### Nutritional status of children

Nutritional status (stunting) among children under the age of five years in this study was 37.7%.

### Factors associated with stunting

Factors associated with stunting in the bivariable analysis include the age of the child, family size, number of under-five children in the household, sex of the caretaker, educational status of the caretaker, occupational status of the caretaker, head of the household, wealth status, birth order, antenatal care followup, age of the child during initiation of the complementary feeding, frequency of complementary feeding, status of colostrum, child vaccinated, child morbidity, source of drinking water, hand washing practice after latrine, waste disposal method, access to diversified diet, and household food security status. After adjusting the effect of confounding variables, age of the child, family size, number of under-five children in the household, wealth status of the household, source of the drinking water, access to diversified diet, and household food security status as major determinants of stunting among children under the age five years in this study area. According to the result of this study, the peak age range for stunting was 36 to 47 months, and then decline for the higher age group (between 48 to 59 months) was exhibited. Children in age groups of less than six months and 6 to 11 months were less likely to be stunted than those above 24 months, [AOR = 0.034, 95% CI: (0.005, 0.24)] and [AOR = 0.174, 95% CI: (0.075, 0.4)], respectively.

Odds of presenting as stunted among those who are living in a household with a household of below five people is less than those living in a household with a family member of five or more [AOR = 0.59, 95% CI: (0.37, 0.97)]. There is an increased risk for stunting in children under five years of age who are living in a household with two or more children under five years of age than those living with only one [AOR = 1.72, 95% CI: (1.07, 2.77)].

Odds of stunting among children under five years of age living in a rich household were less than living in a poor household [AOR = 0.46, 95% CI: (0.266, 0.79)].

The likelihood of being stunted among those who got drinking water from a river, a pond, or a spring is higher than those whose drinking water source was a public tap [AOR = 5.11, 95% CI:1.6, 16.4)], [AOR = 6.24, 95% CI: (2.45, 16)] and [AOR = 2.08, 95% CI: (1.15, 3.75)] respectively.

Children under five years of age who are living in a household with undiversified diet are more likely to be stunted than their counterparts [AOR = 1.82; 95% CI: (1.17, 2.83)].

Children under five years of age living in a food-insecure household were more likely to be stunted when compared to those living in a food secured household [AOR = 1.83; 95% CI: (1.13, 2.96)] (***Table 5***).

**Table 5:** Factors associated with stunting among children under five years of age in Wonago district, December, 2018 (n = 615).


VARIABLES	CATEGORY	STUNTING		OR (95% CI)	
	
		YES FREQUENCY (%)	NO FREQUENCY (%)	CRUDE	ADJUSTED

Age of the child	<6 months	3 (4.2%)	69 (95.8%)	0.05 (0.016, 0.76)***	0.03 (.05,0 .24)**

	6–11 months	18 (24.3%)	56 (75.7%)	0.38 (0.21, 0.67)**	0.17 (0.07, 0.40)***

	12–24 months	65 (43.0%)	86 (57.0%)	0.89 (0.60, 1.32)	0.76 (0.47, 1.22)

	>24 months	146 (45.9%)	172 (54.1%)	1	1

Family size	<5 members	82 (35.2%)	151 (64.8%)	0.65 (0.45, 0.94)*	0.59 (0.37, 0.97)*

	≥5 members	150 (39.3%)	232 (60.7%)	1	1

Number of under-five children in the household	1	135 (39%)	211 (61%)	1	1

	≥ 2	172 (63.9%)	97 (36.1%)	1.53 (1.36, 1.87)**	1.72 (1.07, 2.77)*

Wealth status	Poor	99 (44.6%)	123 (55.4%)	1	1

	Medium	81 (35.7%)	146 (64.3%)	0.80 (0.62, 1.05)	0.89 (0.50, 1.58)

	Rich	52 (31.3%)	114 (68.7%)	0.56 (0.42, 0.73)***	0.46 (0.26,0 .79)**

Source of drinking water	River water	11 (57.9%)	8 (42.1%)	6.18 (1.5, 25.5)*	5.11 (1.6, 16.4)**

	Pond	28 (54.9%)	23 (45.1%)	5.5 (1.63, 18.5)**	6.24 (2.45, 16)***

	Spring	85 (38.3%)	137 (61.7%)	2.27 (1.75, 6.91)*	2.08 (1.15, 3.75)*

	Private pipe	8 (18.2%)	14 (81.8%)	3.8 (0.99, 14.6)	0.47 (0.124, 1.77)

	Well	11 (45.8%)	13 (54.2%)	2.79 (0.91, 8.53)	2.18 (0.72, 6.6)

	Public tap	93 (33.6%)	184 (66.4%)	1	1

Access to a diversified diet	Poor	142 (52.0%)	131 (48.0%)	3.0 (2.16, 4.3)***	1.82 (1.17, 2.83)**

	Good	90 (26.3%)	252 (73.7%)	1	1

Household food security	Food unsecured	111 (45.3%)	134 (54.7%)	1.7 (1.22, 2.37)**	1.83 (1.13, 2.96)*

	Food secured	121 (32.7%)	249 (67.3%)	1	1


* P < 0.05, ** P < 0.01, and *** P < 0.001 ^@^ Others: rotto, tankers.

## Discussion

This study revealed that the prevalence of stunting among children under five years of age was 37.7% (95% CI: 33.8%, 41.5%). Age of the child, family size, number of children under five years of age in the household, wealth status of the household, source of drinking water, access to diversified diet, and household food security status were major determinants of stunting.

The prevalence of stunting in this study was 37.7%, which is close to the national and SNNP Regional percentages (reported by EMDHS 2019) of 37% and 36.3%, respectively [20]; the prevalence in this study also compared closely with corresponding studies conducted in Somali region, Kenya (39%) Padanpur (37.7%), and the Bench Maji zone (35.4%) [21,22,23,24]. This similarity may be due to similarity in study setting and age category. However, this prevalence of stunting was lower than the finding of a study conducted in Bule Hora district, Ethiopia (47.6%), Wondo genet (50.3%), Haramaya district (45.8%), Wolaita Sodo (90.3%), Hidabu Abote district (47.6%), Shire Indaselassie (56.6%), Lasta woreda (49.7%), Nepal (47%), Nigeria (47.6%), and India (43%) [25,26,27,28,29,30,31,32,33,34]. The deviation may be due to the difference in study segment, study period, socio-economic characteristics, health service delivery, and/or study area. The prevalence of stunting in this study was higher than the findings of studies conducted in the Sidama zone, Ethiopia (26.6%), Kenya (21.5%), another study in Kenya’s Busia district (13.3%), and China (8.1%) [17,35,36,37]. This variation in prevalence might be due to the difference in sample size, methodological difference, or due to difference in the socioeconomic background of the study participants.

Some of the determinants identified by this study are akin to those identified by a study conducted at public health facilities of the Gedeo zone, Southern Ethiopia [38]. As to the finding of this study, there were significantly reduced odds of being stunted for children aged less than six months and 6 to11 months than those who were aged above 24 months. This finding is in line with the study conducted in Lasta woreda, northern Ethiopia, Wolaita Sodo, Somali region, Kenya Busia district, Nigeria, Nepal, China, and India [22,28,31,32,33,34,36,37]. Starting from this, it can be asserted that the potential of stunting is serious among those children aged below 36 months. This implies that children under five years of age in this age category are likely to be more and irreversibly stunted [24]. This age group is also susceptible to diarrheal disease, intestinal parasites, and other acute infections as well, and this is perhaps why they are more likely to be stunted.

This study also revealed that family size as a significant predictor of stunting. This finding is consistent with the results of studies done in Wolaita Sodo, Bule Hora district, south Ethiopia, Haramaya district, Somali region, Lasta woreda, northern Ethiopia, Shire Indaselassie, Kenya Busia district, and India [22,25,27,28,31,34,36]. The number of children under five years of age living in the household were a risk factor for stunting; that is, two or more children under five years of age living in a household were more likely to be stunted than one child living in a household. This finding is supported by studies conducted in Bench Maji zone, Haramaya district, Somali region, and Kenya Busia district [22,27,36]. This might be due to food and healthcare inaccessibility with higher family size, especially in low-income families. Another possible explanation might be that mothers belonging to households with many more family members, especially children, might not have enough time to care for their children properly.

According to the finding of this study, the odds of stunting among children under five years of age from a rich family was less likely than those from among the poor. This is in agreement with findings from studies conducted in Lasta woreda, northern Ethiopia, Somali region, Nigeria, and India [22,31,33,34]. The possible reason for this could be that families with low economic status experience more economic stress, hence they are more likely to suffer from food insecurity. Particularly, poor families cannot fulfill the nutritional requirements of their children.

Similarly, housholds consuming water from unsafe sources significantly increase the odds of children being stunted than do their counterparts. The finding of this study is in agreement with studies conducted in Bench Maji zone, Wolaita Sodo, Somali region, Shire Indaselassie, India, Bule Hora district, south Ethiopia, Haramaya district, and Kenya Busia district [22,24,25,27,28,34,36]. The reason for this is likely that children under five years of age that drink water from an unsafe source are likely to be undernourished secondary to infections like diarrheal diseases and other comorbidities.

Children under five years of age living in a household that had poor access to a diversified diet and dwelling in a food-insecured household were more likely to became stunted than their counterparts. The finding of this study is in line with studies conducted in Lasta woreda, northern Ethiopia, Vihiga county, Kenya, Somali region, and India [22,31,34,35]. The possible reason for this is that most (73%) of the families in this study were poor and medium in socio-economic status that are immediate causes for under nutrition. Low income housholds are especially vulnerable to changes in food prices. In addition to that, food insecured households do not have the ability to purchase diversified and nutritionally balanced food.

### Strength and Limitations of the study

As a strength, maternal factors were assessed by enquiring only the biological mothers of the children, which could minimize potential recall biases associated with the long memory of the study participants. The nature of the study design neither represents a seasonal variation of nutritional outcomes nor establishes a causal relationship, and some measurements may not be accurate due to subjective responses and recall biases in answers based on the memories of the mothers or the caretakers. This could be raised as a limitation.

## Conclusion and recommendations

According to the WHO global database, the prevalence of stunting among children under five years of age identified by this study in the study area is high [39]. This study revealed that a family size of less than five members, being younger than 11 months old, and living in a rich wealth home contribute to a protective effect against the risk of stunting, while unsafe drinking water sources, the presence of two or more children under five years of age in the household, poor access to diversified diet, and household food insecurity increase the risk of stunting. Efforts should be made to improve the nutritional status of these children through health education; nutrition education about the consumption of a diversified diet from locally available sources; to encourage limiting the number of family members; strengthening the promotion and use of different family planning methods; and to improve the wealth status of women and encourage them to involve and create different income generating activities. Finally, in order to identify the problem early, nutrition surveillance programs such as community health days (CHDs) need to be done regularly. Further longitudinal studies are recommended.

## Data availability statement

All the data included in the manuscript can be accessed from the corresponding author Moges Mareg upon reasonable request through the email address “*metanmann@gmail.com*”.
